# Supplementary material for: The influence of considering individual resistance training variables as a whole on muscle strength: A systematic review and meta-analysis protocol
Source: PLoS One. 2022 Jan 20;17(1):e0262674. doi: 10.1371/journal.pone.0262674 (PMC8775318; doi:10.1371/journal.pone.0262674)
Supplement: Attachment — Submitted filename: Comments.docx [file pone.0262674.s002.docx]

Study Protocol for a Systematic Review with Meta-Analysis: The Influence of Resistance Training Dose, Dosage and Dosing on Muscle Strength

Dear authors, below are my comments. In opinion the work needs major improvement and should not continuing in the review. The work is well written, but the foundation for the execution/reproducibility of the project is little sustaintable. It would be interesting if the authors presented minimal mechanistic bases to justify the analysis of the interaction of variables. There are several studies that analyzed each of the variables in isolation, so why would it be necessary to carry out a combined analysis? What supports this analysis? And why should it be done from the perspective of dose, dosing, and dosage? What each of these analyzes contribute to the training prescription? To illustrate, the authors used more than one page in the introduction to conceptualize the variables (Ln 80-137). This approach is necessary, but brief, being important to substantiate/hypothesize why and how the interaction of these variables allows for a greater increase in strength production after an intervention.

It would be interesting to clarify which are the dependent and independent variables of the study and how the statistical procedures will contribute to the establishment of dose, dosage, and dosage. Finally, I think you should have a specific group for the analysis (adult, non-trainined, trained, atlhetes etc) or a region of the body, or even a specific exercise. The work is very wide and without a direction it may be unlikely to be carried out.

Below there are some points of concern:

Title: The terms dose, dosing, and dosage are confusing, and only explained along the text. I suggest not leaving them in the title.

Ln 38. You wrote sets x repetitions. I think you would like to say: number of sets x number of repetition. Please clarify.

Ln. 24. I did not find te Propero ID (https://www.crd.york.ac.uk/prospero/) by the number provided.

Ln 30 The word style seems to be different.

Ln 33. The pooled standardised mean differences of what?

Ln 34-37. It is necessary to describy what are you comparing.

Ln 37-39. Why do you think the proposed variables are enougth to compose the best prespriction for strength increase? Previous studies indicated the strength increase is specific to the range of motion training (<https://pubmed.ncbi.nlm.nih.gov/23604798/>; <https://pubmed.ncbi.nlm.nih.gov/33977835/>; <https://pubmed.ncbi.nlm.nih.gov/31230110/>). However, nothing is said about the range of motion. If the strength increase is dependent of the range of motion trained, why this variable was not include or even mentioned?

Ln 51.” Resistance training guidelines have been developed to guide”: repetitve terms.

Ln 55. why the other variables were not cited?

Ln 56. The reference cited only informs about volume and intensity. What about the other variables mentioned? They shoud be covered by other references.

Ln 56-58. You present a problem that you are not able to solve by the analysis offered, as this prescription requires the analysis of other variables such as execution speed, range of motion and pause. I recommend you be more conservative.

Ln 66 You describe a problem (which variable is most important for maximal strength increase...) that you won't be able to answer. I suggest rephrasing the sentence.

Ln 71-73. The analysis of volume x intensity allows to understand the dose of the training section. The analysis of volume x intensity x frequency allows us to understand the weekly dose, but the analysis of these variables with duration would allow which interpretation? nothing was said about it?

Ln 76. “must consider the duration of a program”. Why? Please, you need to clarify these issue. What about other variable that were left out, such as workload? (https://pubmed.ncbi.nlm.nih.gov/21113614/)

Ln 80 a 137. The concepts of each variable could have been summarized in two paragraphs, and then, the reasoning of the neurophysiological mechanisms that support the importance of analyzing the interaction of the variables presented in the force response could have been presented.

Ln 111. “It is also important to consider the influence of RT frequency and duration” . Why it is importante to consider? Please clarify

Ln 115-117. No reference was given.

Ln 122. It is importante to metion that it is account only for the same muscle group.

Ln 135-137. Little information was given to “Duration” compared to the other variables.

Ln 149. The question presented has not yet been answered and will not be answered by the study. I see no justification for your permanence. Perhaps present a question closer to the question you want to answer.

Ln 154-158. Please clarify how the dose, dosing and dosage could influence the development of muscle strength.

Ln 174. “All” articles prior to july 2021? I think you should delimitated a specific group (i.e., https://www.ncbi.nlm.nih.gov/pmc/articles/PMC4656698/), or a body member, or an specific exercise.

Ln 178. Which groups?

Ln 235. Please, give an example for better understanding. Why the median and not the mean?

Ln 245-248. Why 10%? What is the rationale for this procedure?

Ln 268. No answer is absolute. Each study adds knowledge on the subject and its contribution cannot be disregarded. Please re-do the sentence.

Ln 269. “Instead, previous analyses have simply categorised variables/outcomes to 270 identify if one category is different to another “ . As it is, it gives the impression that the previous work was reduced and contributed little. I believe that the phrase could be redone by valuing the previous work and at the same time adding actions that would complement the previous initiative.

Ln 271-272. You are disregarding the other variables. I think the phrase would be better if you commented only for the effect of the interaction between the variables that are being investigated.
